# Optimization of Fluorescent Tools for Cell Biology Studies in Gram-Positive Bacteria

**DOI:** 10.1371/journal.pone.0113796

**Published:** 2014-12-02

**Authors:** Maria João Catalão, Joana Figueiredo, Mafalda X. Henriques, João Paulo Gomes, Sérgio R. Filipe

**Affiliations:** 1 Laboratory of Bacterial Cell Surfaces and Pathogenesis, Instituto de Tecnologia Química e Biológica, Universidade Nova de Lisboa, Avenida da República, Apartado 127, 2781-901 Oeiras, Portugal; 2 National Institute of Health, Department of Infectious Diseases, Av. Padre Cruz, 1649-016 Lisbon, Portugal; University Medical Center Utrecht, The Netherlands

## Abstract

The understanding of how Gram-positive bacteria divide and ensure the correct localization of different molecular machineries, such as those involved in the synthesis of the bacterial cell surface, is crucial to design strategies to fight bacterial infections. In order to determine the correct subcellular localization of fluorescent proteins in *Streptococcus pneumoniae*, we have previously described tools to express derivatives of four fluorescent proteins, mCherry, Citrine, CFP and GFP, to levels that allow visualization by fluorescence microscopy, by fusing the first ten amino acids of the *S. pneumoniae* protein Wze (the i-tag), upstream of the fluorescent protein. Here, we report that these tools can also be used in other Gram-positive bacteria, namely *Lactococcus lactis*, *Staphylococcus aureus* and *Bacillus subtilis*, possibly due to optimized translation rates. Additionally, we have optimized the i-tag by testing the effect of the first ten amino acids of other pneumococcal proteins in the increased expression of the fluorescent protein Citrine. We found that manipulating the structure and stability of the 5′ end of the mRNA molecule, which may influence the accessibility of the ribosome, is determinant to ensure the expression of a strong fluorescent signal.

## Introduction

*Streptococcus pneumoniae* is a Gram-positive bacterium usually found in association with a range of different types of infections. It is a common respiratory pathogen, capable of causing low severity otitis media or more serious infections such as pneumonia or meningitis, as well as a frequent cause of community-acquired pneumonia in developed countries [1].

Pneumococcal bacteria can colonize the mucosal surface of the upper respiratory tract, while remaining undetected and asymptomatic [2]. However, when pneumococcal bacteria gain access to normally sterile locations of the organism, they are capable of successfully propagating, in spite of the different defence mechanisms of the host immune system [1].

The understanding of how bacteria divide or perform specific tasks important for their survival inside the host is a requirement for the design of efficient strategies to fight bacterial infections. This implies a detailed knowledge not only of the function of proteins required for the infection process, but also of their localization and role in complex molecular machineries. The availability of genetic and cell biology tools that allow controlled expression of proteins of interest, as well as the study of their localization, is therefore particularly important for the study of bacterial pathogens.

We have recently described tools that can contribute to the study of the pneumococcal biology by ensuring the expression of fusions of *S. pneumoniae* proteins to different fluorescent proteins, namely mCherry, Citrine, CFP and GFP [3]. This was achieved through the introduction of an upstream tag, named “i-tag”, which increased the efficiency of protein translation.

We have now constructed different plasmids to demonstrate that this tag is also efficient in increasing expression of fluorescent proteins in different Gram-positive bacteria, such as *Lactococcus lactis*, *Bacillus subtilis* and *Staphylococcus aureus*.

In order to understand how the efficiency of protein translation is improved, we have determined the molecular requirements of the sequence encoding the i-tag that ensure the expression of the fluorescent proteins. We have identified additional 10 amino acid tags, derived from different pneumococcal proteins, which could also increase expression of fluorescent proteins by decreasing of the stability of the structure of 5′ end of the transcribed mRNA molecule.

## Results and Discussion

### The first ten amino acids of the protein Wze, named the i-tag, can increase the expression of the Citrine fluorescent signal in different bacterial models

We have previously described the construction of a set of plasmids for the expression of fluorescent protein fusions in the low-GC Gram-positive *S. pneumoniae* bacteria [3]. These plasmids were designed in order to improve expression of the fluorescent proteins mCherry, Citrine, CFP and GFP by including a sequence encoding the first ten amino acids of the capsular protein Wze, which we named the i-tag, upstream of the fluorescent proteins. We proposed that this i-tag extension might facilitate ribosome accessibility to the ribosome-binding site, thus enhancing protein translation [3].

In order to determine if we could apply a similar strategy to other bacterial models, we inquired whether the i-tag could likewise increase expression of fluorescent proteins in other Gram-positive bacteria, namely *Lactococcus lactis*, *Staphylococcus aureus* and *Bacillus subtilis*.

**Figure 1:**
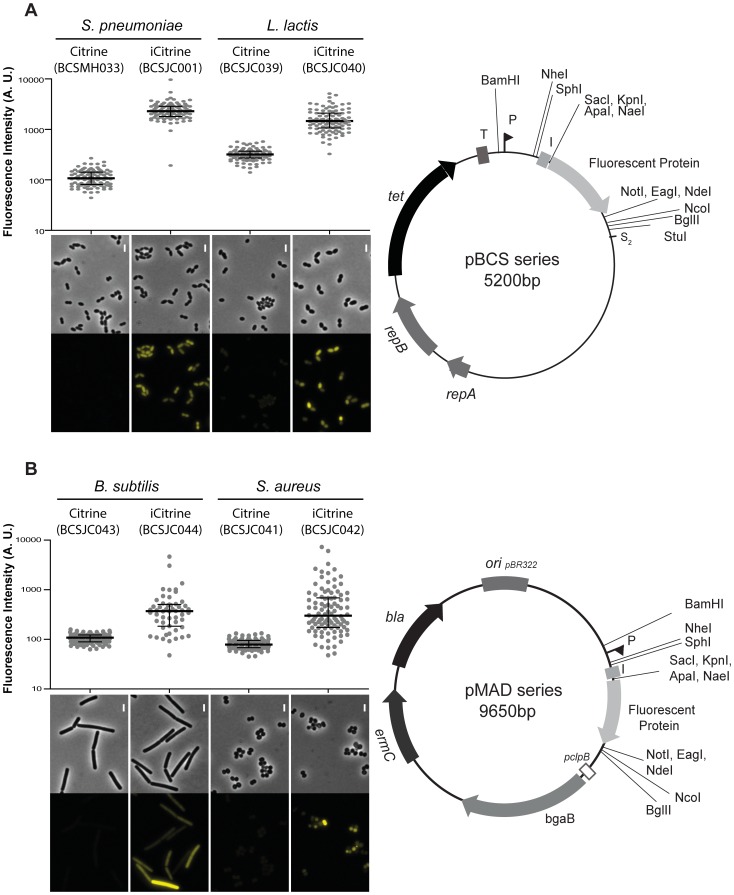
Linking the i-tag to Citrine improves the expression of fluorescence in Gram-positive bacteria. **A**) (Left panel) Median fluorescence, with 25% and 75% inter-quartile range (black lines) of the fluorescence signal detected in *S. pneumoniae* unencapsulated bacteria, in arbitrary units (A. U.), expressing Citrine (strain BCSMH033) or iCitrine (strain BCSJC001), and *L. lactis*, expressing Citrine (strain BCSJC039) and iCitrine (strain BCSJC040). At least 100 cells of each strain were quantified. Representative images of each strain are shown below the graph. Scale bar: 1 µm. (Right panel) Map of the pBCS plasmids. Fluorescent protein refers to Citrine and iCitrine. *repA*, *repB*, plasmid replication genes. *tet*, tetracycline resistance marker. T, transcription terminator. P, promoter. S2, stop codon. **B**) (Left panel) Median fluorescence, with 25% and 75% inter-quartile range (black lines) of the fluorescence signal emitted by Citrine and iCitrine detected in *S. aureus* bacteria, in arbitrary units (A. U.), expressing Citrine (strain BCSJC041) and iCitrine (strain BCSJC042), or *B. subtilis*, expressing Citrine (strain BCSJC043) or iCitrine (strain BCSJC044). At least 100 cells of each strain were quantified. Representative images of each strain are shown below the graph. Scale bar: 1 µm. (Right panel) Map of the pMAD plasmids. Fluorescent protein refers to Citrine and iCitrine. Unique restriction sites are indicated. *ermC*, erythromycin resistance marker.

We transformed *L. lactis* with the previously constructed plasmid pBCSJC001, which allows the expression of the improved form of the fluorescent protein Citrine (iCitrine) [3], and with pBCSMH002, which should allow expression of Citrine fusion protein without any tag [4]. These vectors are derivatives of the plasmid pLS1 that was shown to efficiently replicate and confer tetracycline resistance in Gram-positive bacteria [5] including *L. lactis* [6]. Quantification of the fluorescent signal expressed by *L. lactis* strains encoding Citrine alone (BCSJC039) or the improved form of Citrine (BCSJC040), showed that, as formerly observed for *S. pneumoniae* [3] the fusion of the first ten amino acids of Wze (i-tag) increased expression of fluorescence. *L. lactis* strain BCSJC039, expressing only the untagged fluorescent protein Citrine, showed low levels of fluorescence (Fig. 1A). We then enquired whether the i-tag could likewise increase expression of fluorescent proteins in other Gram-positive bacteria besides *L. lactis.* As we did not succeed to transform these plasmids into other Gram-positive bacteria, we cloned the DNA fragment containing both the promoter and the coding sequence of the fluorescent proteins into the pMAD vector, which could be transformed into other Gram-positive bacteria. The pMAD plasmid contains a termosensitive replication origin and replicates at 30°C degrees [7].

We constructed two additional vectors by cloning the conserved −10 constitutive promoter region of *SigA* from *S. pneumoniae*, the ribosome-binding site and Citrine (from plasmid pBCSMH002) or iCitrine (from plasmid pBCSJC001) in pMAD, which generated plasmids pBCSJC039 and pBCSJC040, respectively. The resulting plasmids were transformed in *S. aureus* and *B. subtilis* originating strains BCSJC041 to BCSJC044 and Citrine expression was detected by fluorescence microscopy. We observed an increase in fluorescence intensity only when the i-tag was fused to Citrine both in *S. aureus* strain BCSJC042 and *B. subtilis* strain BCSJC044. Heterogeneity in fluorescence intensity was observed in both bacterial species, which may be due to plasmid copy number instability at 30°C degrees even under the selective pressure of erythromycin antibiotic. The observed heterogeneity may be reduced using other replicative plasmids or by expressing the fluorescent proteins from the chromosome. Low levels of fluorescence were detected when Citrine (without the i-tag) was expressed in *S. aureus* BCSJC041 and *B. subtilis* BCSJC043 strains (Fig. 1B).

Therefore, as observed for *S. pneumoniae* [3], the presence of the nucleotide sequence encoding the i-tag upstream of the fluorescent protein Citrine results in increased fluorescence intensity, possibly due to optimized translation rates, in the Gram-positive bacteria *L. lactis*, *S. aureus* and *B. subtilis*.

### Determination of the N-terminal tag requirements to improve expression of fluorescent proteins in *Streptococcus pneumoniae*

**Figure 2:**
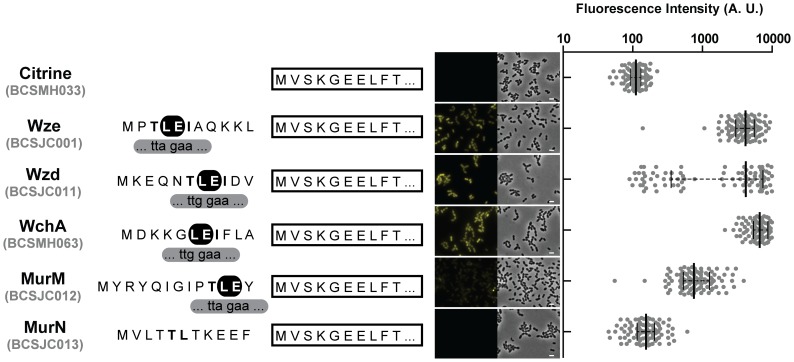
The amino terminal end of different *S. pneumoniae* proteins ensures expression of the Citrine fluorescent signal when a conserved LE motif is present. (Left panel) Sequence of the first aminoacids of proteins WchA, MurM, MurN, Wze and Wzd that were linked to Citrine (shown as a white rectangle) are shown. Highlighted (black) are the conserved aminoacids L (leucine) and E (glutamic acid). (Right panel) Median fluorescence, with 25% and 75% inter-quartile range (black lines) of the fluorescence signal detected in *S. pneumoniae* R36A unencapsulated bacteria, in arbitrary units (A. U.), emitted by Citrine (BCSMH033), WchA_(1-10)_-Citrine (BCSMH063), MurM_(1-10)_-Citrine (BCSJC012), MurN_(1-10)_-Citrine (BCSJC013), Wze_(1-10)_-Citrine (BCSJC001) and Wzd_(1-10)_-Citrine (BCSJC011). At least 100 cells of each strain were quantified. Representative images of each strain are shown. Scale bar: 1 µm.

In order to determine how the sequence of the i-tag permitted an efficient expression of the fluorescent proteins, we decided to investigate if the N-terminal of other highly expressed pneumococcal capsular proteins were also capable of increasing the expression of fluorescent proteins in *S. pneumoniae*. We had observed a strong fluorescent signal when some of these proteins had their C-terminus end fused to the Citrine fluorescent protein [3]. For this purpose, we initially redesigned plasmid pBCSJC001 and substituted the i-tag encoding the first ten amino acids of Wze, for the sequences encoding the first ten amino acids of Wzd (originating plasmid pBCSJC011) and the first eleven aminoacids of WchA (originating plasmid pBCSMH061), two proteins involved in capsular biosynthesis [8]. These plasmids were transformed in *S. pneumoniae* R36A originating strains BCSJC011 and BCSMH063, which expressed Citrine with a N-terminal tag constituted by 10/11 amino acids of Wzd or WchA, respectively. When protein expression was determined by fluorescence microscopy, we noticed that the fluorescence signal in strain BCSMH063, encoding WchA_10aa_-Citrine, was higher than the signal observed in strain BCSJC001 strain, encoding the original iCitrine fusion, Wze_10aa_-Citrine (Fig. 2). The expression of Wzd_10aa_-Citrine also resulted in higher fluorescent signal but in this case only in a sub-population of cells (Fig. 2).

We also designed two additional plasmids in which the fluorescent protein Citrine was fused, at its N-terminus, to the first amino acids of MurM (pBCSJC012) or MurN (pBCSJC013), two *S. pneumoniae* enzymes that are involved in the synthesis of a branched peptidoglycan [9], originating strains BCSJC012 and BCSJC013, respectively. Fluorescence microscopy analysis showed that in both cases, the expression of fluorescence was lower than for iCitrine. This was particularly noticeable for the expression of MurN_10aa_-Citrine fusion, which did not result in fluorescent bacteria (Fig. 2).

Amino acid sequence alignment of the N-terminal regions of Wze, Wzd, WchA, MurM and MurN showed that a motif of two amino acids, a leucine (L) and a glutamate (E), are conserved in those that allowed the expression of higher fluorescent signals (Wze, Wzd, WchA and MurM). However, neither the leucine codon (UUG or UUA) nor its distance to the start codon was conserved (Fig. 2).

**Figure 3:**
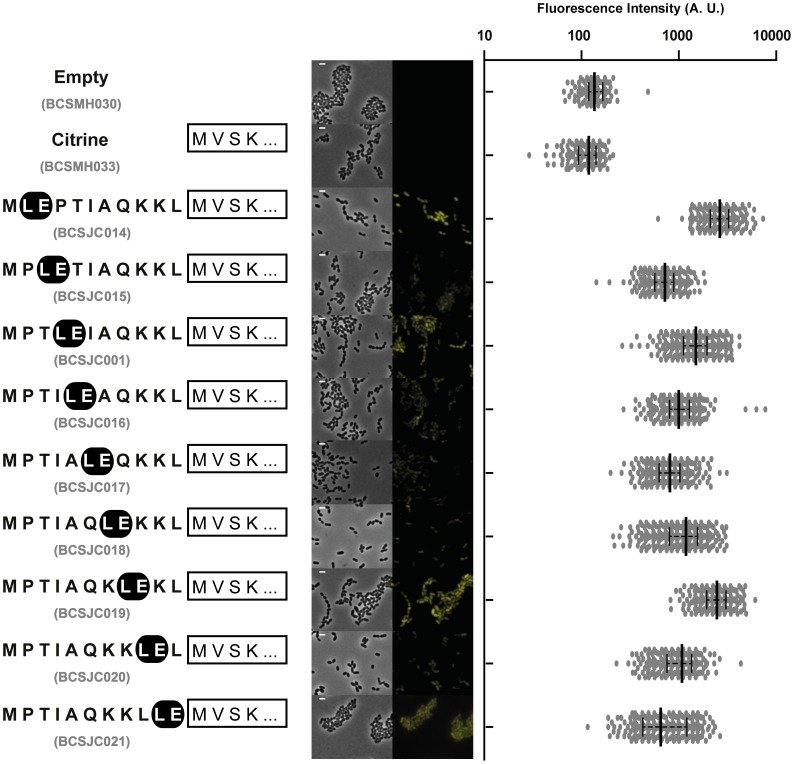
Expression of the Citrine fluorescent signal is not dependent on the distance of the conserved LE motif to its N-terminal end. Aminoacid sequence of the different tags, containing the LE motif successively positioned 0 to 9 amino acids distant from the starting methionine, linked to Citrine (shown as a white rectangle) is shown. Median fluorescence, with 25% and 75% inter-quartile range (black lines) of the fluorescence signal emitted by the following unencapsulated bacteria *S. pneumoniae* R36A strains, in arbitrary units (A. U.): Empty plasmid (BCSMH030), Citrine (BCSMH033), i*(MLEPTIAQKKL)-Citrine (BCSJC014), i*(MPLETIAQKKL)-Citrine (BCSJC015), i*(MPTLEIAQKKL)-Citrine (BCSJC001), i*(MPTILEAQKKL)-Citrine (BCSJC016), i*(MPTIALEQKKL)-Citrine (BCSJC017), i*(MPTIAQLEKKL)-Citrine (BCSJC018), i*(MPTIAQKLEKL)-Citrine (BCSJC019), i*(MPTIAQKKLEL)-Citrine (BCSJC020), and i*(MPTIAQKKLLE)-Citrine (BCSJC021). The strains BCSMH030 and BCSMH033 were used as a negative control. At least 100 cells of each strain were quantified. Representative images of each strain are shown. Scale bar: 1 µm.

In order to determine the optimal distance of this conserved LE motif to the start codon, we constructed a set of plasmids (pBCSJC014 to pBCSJC021) in which the LE encoding codons were 0 to 9 codons away from the start codon (Fig. 3). *S. pneumoniae* R36A strains BCSJC014 to BCSJC021 expressing these plasmids were analysed by fluorescence microscopy. The highest fluorescent signal was observed for BCSJC014 and BCSJC019 strains, in which the LE motif was located immediately next to or six amino acids far from the starting methionine, respectively (Fig. 3). In all other cases, fluorescence levels were about 50% lower than for iCitrine (Fig. 3). Therefore the distance of the LE motif to the N-terminal end of the protein was not a crucial factor to ensure increased expression of Citrine.

As the Leucine codon also varied in the different N-terminal regions that were tested (Fig. 2), we speculated whether the use of less frequently used codons could prevent the successful expression of the Citrine fluorescent protein, as the introduction of less frequently used codons in the beginning of mRNA molecules may result in the reduction of the translation of the encoded proteins. This hypothesis had been previously suggested for *B.* subtilis, where a sequence encoding the first eight aminoacids of specific ComGA was proposed to overcome the slow translation initiation caused by the eukaryotic codon bias present in fluorescent proteins [10]. This is due to the fact that the absence of tRNA molecules can result in a stalled translation process, which consequently may lead to the disassembly of the complex ribosome/mRNA [11] [12].

**Figure 4:**
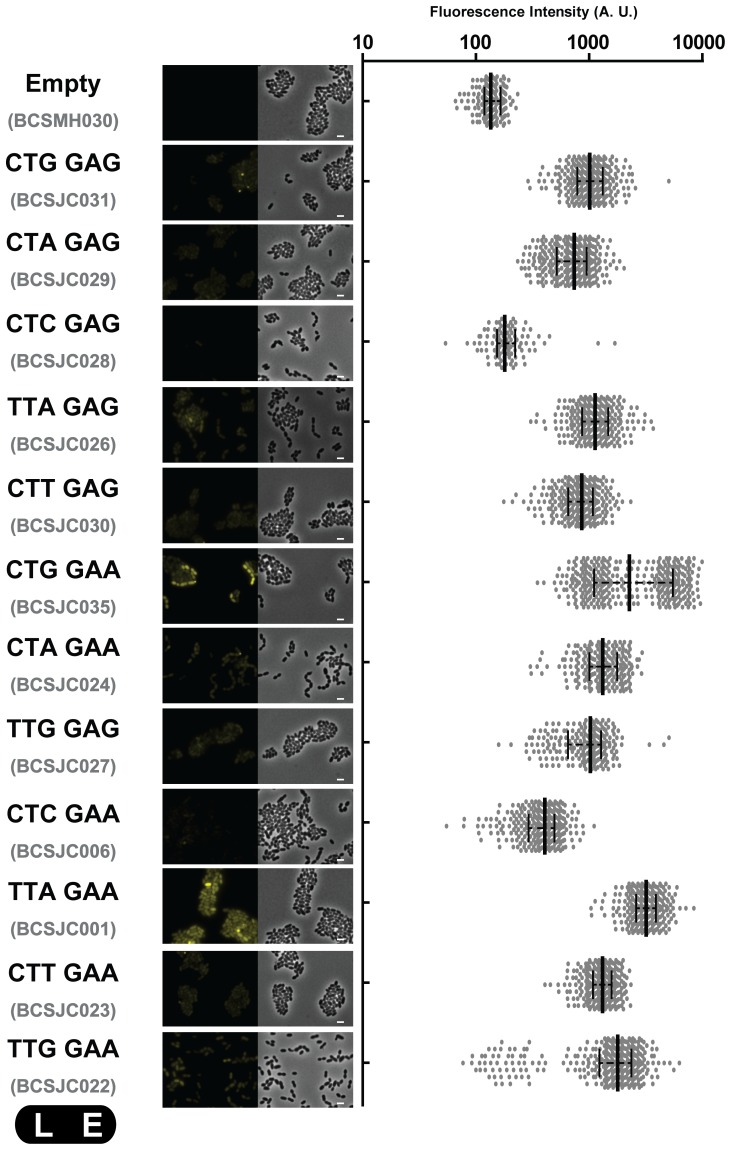
Fluorescence intensity is not only dependent on codon usage. Different pBCSJC001 derivative plasmids were constructed where the leucine codon in the fourth position of the i-tag (TTA) was replaced by the alternative leucine codons (TTG, CTT, CTA, CTG), and/or the glutamate codon in the fifth position of the i-tag (GAA) was replaced by the alternative glutamate codon (GAG). Median fluorescence, with 25% and 75% inter-quartile range (black lines) of the fluorescence signal emitted by the following unencapsulated bacteria *S. pneumoniae* R36A strains, in arbitrary units (A. U.): Empty plasmid (strain BCSMH030 used as a negative control), CTGGAG (frequency of 0.0002, strain BCSJC031), CTAGAG (frequency of 0.0002, strain BCSJC029), CTCGAG (frequency of 0.0003, strain BCSJC028), TTAGAG (frequency of 0.0004, strain BCSJC026), CTTGAG (frequency of 0.0004, strain BCSJC030), CTGGAA (frequency of 0.0005, strain BCSJC025), CTAGAA (frequency codon usage of 0.0006, strain BCSJC024), TTGGAG (frequency of 0.0006, strain BCSJC027), CTCGAA (frequency of 0.0007, strain BCSJC006), TTAGAA (frequency of 0.0010, strain BCSJC001), CTTGAA (frequency of 0.0011, strain BCSJC023) and TTGGAA (frequency of 0.0015, strain BCSJC022). At least 100 cells of each strain were quantified. Representative images of each strain are shown. Scale bar: 1 µm.

We therefore constructed different pBCSJC001 derivative plasmids (pBCSJC006, pBCSJC022 to pBCSJC025), where the leucine codon in the fourth position of the i-tag (UUA, codon usage frequency of 0.0198) was replaced by the alternative leucine codons, which are predicted to be differently used by *S. pneumoniae* CUG (0.0092), CUA(0.0116), CUC (0.0127), CUU(0.0207) and UUG(0.0287) [13]. Pneumococcal strains carrying the iCitrine fusion expressing these silent mutations were observed by fluorescence microscopy and the fluorescent signal obtained was quantified. The highest fluorescence signal was obtained for the strain expressing the original iCitrine fusion where leucine is coded by the UUA codon (BCSJC001) (Fig. 4). Interestingly, we also observed high fluorescence signals for the strains expressing the modified iCitrine fusion where the UUA leucine codon was replaced by either the lowest frequency leucine CUG (BCSJC025) or the highest frequency leucine codon UUG (BCSJC022), indicating that codon usage does not determine the efficiency of the i-tag.

We also tested the effect of exchanging the glutamate codon in the fifth position of the i-tag (GAA, codon usage frequency of 0.0517) for the alternative glutamate codon (GAG, codon usage frequency of 0.0211), which resulted in lower levels of fluorescent signal (Fig. 4).

The observation that the combination of the less frequently used codons or of the more frequently used codons did not always result in non-fluorescent or highly fluorescent pneumococcal bacteria, respectively, allowed us to conclude that fluorescence stabilization is codon-sequence specific in a way that seems not to be influenced by the usage of less frequently-used codons.

### Fluorescence intensity seems to be dependent on the stability of the 5′ end of the mRNA structure

**Figure 5:**
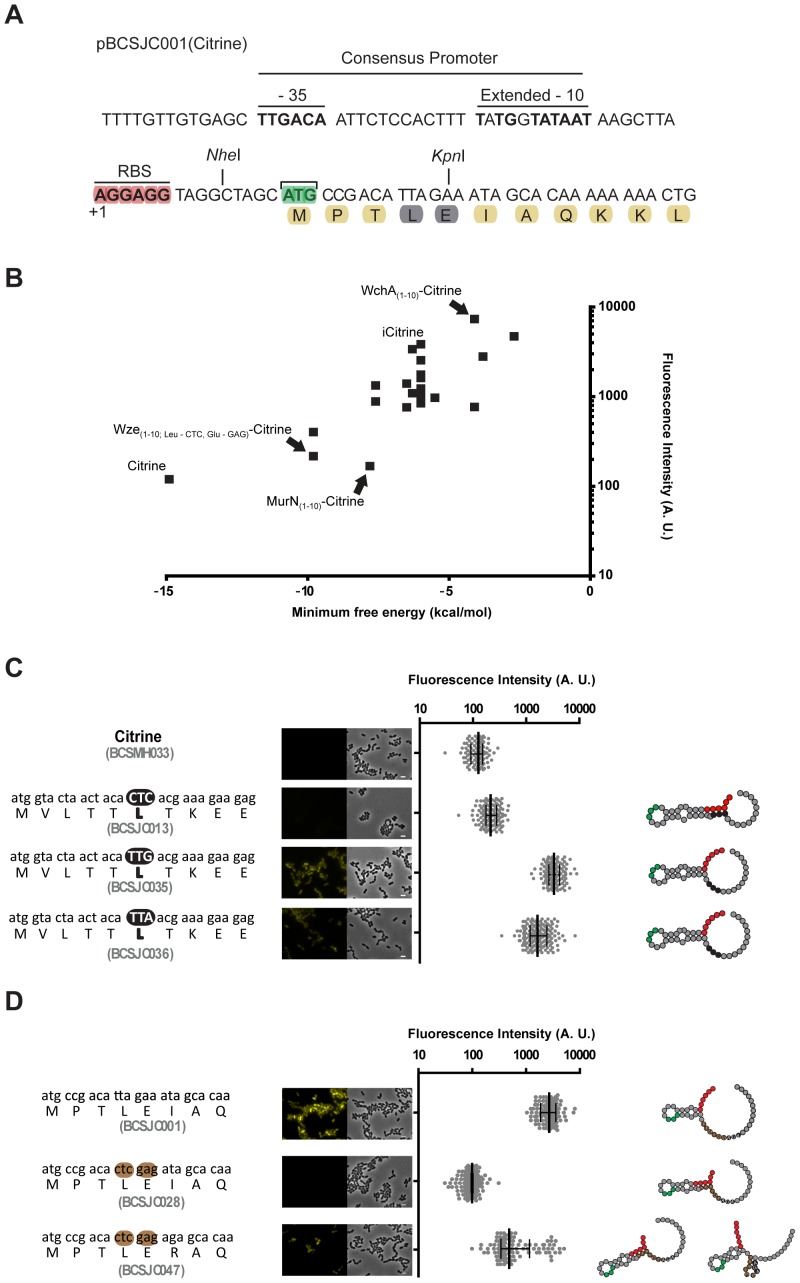
Expression of Citrine derivatives containing N-terminal tags is dependent on ribosome-binding site accessibility. (**A**) Partial sequence of plasmid pBCSJC001 highlights the consensus promoter region, the ribossome-binding site, the proposed transcription start site (+1) and translation start site (AUG) [18] [19]. (**B**) The mean fluorescence measured for the different constructs where Citrine has been fused to different tags at its N-terminal end is plotted relatively to the predicted thermal stability (minimum free energy in kcal/mol) of the 5′ end of the mRNA (+1 to +45) structures. The values for some Citrine derivatives are highlighted: Citrine (expressed in strain BCSMH033), iCitrine (strain BCSJC001), WchA_(1-10)_-Citrine (strain BCSMH063), MurN_(1-10)_-Citrine (strain BCSJC012) and Wze_(1-10)_-Citrine with mutated leucine (CTC) and glutamate (GAG) codons (strain BCSJC028). (**C**) Replacing the wild-type CTC leucine codon in pBCSJC006 for the alternative TTG and TTA codons in the tag derived from the N-terminal end of MurN resulted in increased cell fluorescence. Median fluorescence, with 25% and 75% inter-quartile range (black lines) emitted by unencapsulated R36A *S. pneumoniae* strains expressing Citrine (used as a negative control, strain BCSMH033), MurN_(1-10)_-Citrine with CTC codon (strain BCSJC013), with TTG codon (strain BCSJC035) and with TTA (strain BCSJC036). At least 100 cells of each strain were quantified. (Left) Representative images of each strain as well as the peptide and nucleotide sequences of the N-terminal tag. Scale bar: 1 µm. (Right) Representation of the 5′-end mRNA structure. Ribosome binding site (Red), AUG codon (Green) and mutated nucleotides (black) are highlighted. (**D**) Mutating the ATA isoleucine codon in pBCSJC028 to the AGA arginine codon in the tag derived from the N-terminal end of Wze resulted in increased cell fluorescence. Median fluorescence, with 25% and 75% inter-quartile range (black lines) emitted by unencapsulated R36A *S. pneumoniae* strains expressing iCitrine (used as a positive control, strain BCSJC011), Wze_(1-10)_-Citrine with CTC leucine and glutamate GAG codons (strain BCSJC028), Wze_(1-10)_-Citrine with CTC leucine, glutamate GAG and arginine AGA codons (strain BCSJC047). At least 100 cells of each strain were quantified. (Left) Representative images of each strain as well as the peptide and nucleotide sequences of the N-terminal tag. Scale bar: 1 µm. (Right) Representation of the 5′-end mRNA structure. Ribosome binding site (Red), AUG codon (Green) and mutated nucleotides (black) are highlighted.

We hypothesized that the effect of N-terminal i-tag on the expression of the Citrine protein could be related to the structure of the 5′ end of the mRNA molecule produced or to its stability, which consequently could improve the accessibility of the ribosome. Therefore, the 5′ ends of the mRNAs originated by the introduction of the different tags described above were evaluated by the RNAfold program available at the ViennaRNA Web Services [14]. When we plotted the intensity of the fluorescent signal detected in the pneumococcal strains expressing Citrine with different N-terminal ends, with the respective minimum free energy predicted for the 5′ end of the corresponding mRNA molecules (Fig. 5A), we observed an apparent inverse correlation between the stability of mRNA structures and the intensity of the fluorescent signals (Fig. 5B). The highest fluorescent signal was observed with strain BCSMH063 that expressed the WchA_10aa_-Citrine.

We also noticed that in the strains with the lowest levels of fluorescence, such as that encoding MurN_10aa_-Citrine, the AGGAGG ribosome-binding site was stably paired with the leucine codon (Fig. 5C) possibly impairing ribosome binding to the mRNA molecule. Therefore, we asked whether replacing the leucine codon for an alternative codon, which would not pair with the RBS and would decrease the stability of the predicted structure of the 5′end of the mRNA, could allow protein translation initiation and the expression of a fluorescent signal. For this, we constructed plasmids pBCSJC35 and pBCSJC036 in which the *murN* wild-type CUC leucine codon, present in plasmid pBCSJC013, was substituted by the alternative leucine codons (the less frequently used UUG and the more frequently used UUA, respectively). As expected, resulting strains BCSJC035 and BCSJC036 showed higher levels of fluorescence (Fig. 5C).

Similar results could be obtained by mutating the codon located next to the leucine CUC and glutamate GAG codons in the iCitrine expressed by BCSJC028 strain. Although the presence of these codons prevented the expression of any fluorescent signal, mutating the codon next to them, from AUA to AGA, which converts an isoleucine to an arginine, resulted in increased fluorescence in accordance with the predicted decrease in the stability the 5′end mRNA structure and the improved accessibility to the ribosome binding site (Fig 5D).

**Figure 6:**
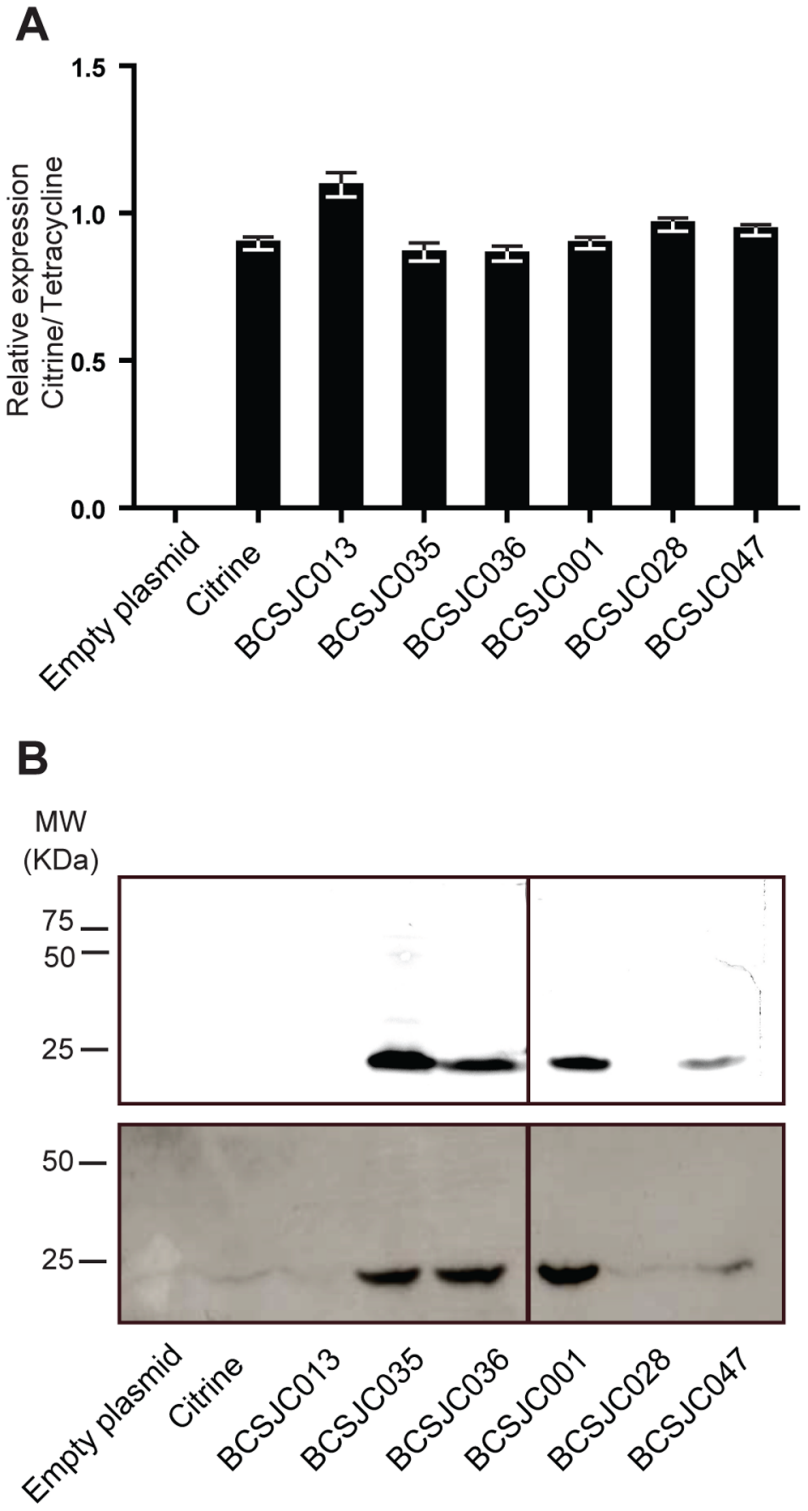
Increased fluorescence resulting from the presence of different tags is due to increased protein levels. (**A**) mRNA encoding Citrine was quantified by qPCR in strains expressing specific aminoacid sequences from different tags fused to Citrine, relatively to the mRNA for the tetracycline resistance protein which is encoded in the plasmid backbone. Strains analyzed were BCSMH030 (transformed with an empty vector), BCSJC013 (expressing MurN_10aa__Citrine), BCSJC035 (expressing MurN_10aa(CTC-TTG)_-Citrine), BCSJC036 (expressing MurN_10aa(CTC-TTA)_-Citrine), BCSJC001 (expressing Wze_(1–10)_-Citrine), BCSJC028 (expressing Wze_10aa(TTA-CTC, GAA-GAG)_-Citrine), BCSJC047 (expressing expressing Wze_10aa(TTA-CTC, GAA-GAG, ATA-AGA)_-Citrine). (**B**)Cell extracts from these strains were separated by SDS-Page and analyzed using a Fluorescent Image Analyzer (**top panel**) and by Western-blot analysis using an antibody that recognizes Citrine protein (**bottom panel**), showing that Citrine fluorescence results from increased protein levels and not increased mRNA levels.

In order to determine if the increased fluorescence observed for strains BCSJC035, BCSJC036 and BCSJC047 could result from higher mRNA levels or higher protein translation, we quantified by qPCR, also known as real-time PCR the levels of mRNA of these strains in exponentially growing bacteria, relatively to the mRNA of the tetracycline resistance marker, encoded in the plasmid backbone. We observed that levels of the different mRNAs were not sufficiently different to explain the variability in fluorescence expression amongst the fluorescent and non-fluorescent strains (Fig. 6A). Citrine protein levels were also quantified using a fluorescent image analyzer and Western-blot analysis. We observed that Citrine could be detected only in fluorescent strains, namely those encoding for MurN_CTC-TTG_-Citrine (strain BCSJC035), MurN_CTC-TTA_-Citrine (strain BCSJC036), MurM_CTC-GAG-AGA_-Citrine (strain BCSJC47) and strain Wze_10aa_-Citrine (strain BCSJC001 which encodes iCitrine) (Fig. 6B). Taken together these results show that the stability of the 5′end of the transcribed mRNA, and the pairing with ribosome-binding site, which may prevent the access of the ribosome, determine the efficiency of the expression of Citrine derivatives in *S. pneumoniae*.

### Final Remarks

The study of protein localization in different bacterial species requires the development of flexible tools. Here we have shown that tools previously developed for the Gram-positive pathogen *S. pneumoniae*, can be used in other pathogenic bacteria, namely *S. aureus*, in the model organism *B. subtilis* or in *L. lactis*, a species of great industrial interest, suggesting that these tools can have a widespread application.

Furthermore, we have optimized the sequence of the i-tag in order to maximize the production of the Citrine fluorescent protein in *S. pneumoniae* by manipulating the structure and stability of the mRNA 5′ end encoded by the i-tag. The 5′ end of mRNA transcripts plays a major role in the translation initiation and determines the translation rate and consequently the total amount of protein produced [15]. We propose that the determination of minimum free energy predicted for the 5′ end of the mRNAs molecules originated by the introduction of the different tags may predict whether the expression of a specific fluorescent protein will be successful.

## Materials and Methods

### Bacterial strains and growth conditions

All bacterial strains and plasmids used in this study are listed in Table 1. *Streptococcus pneumoniae* was grown in C + Y liquid medium [16] at 37°C, without aeration, or in tryptic soy agar (TSA, Difco) plates supplemented with 5% sheep blood (Probiologica). Tetracycline was added to the media at 1 µg/ml final concentration. *Bacillus subtilis* was grown in Luria-Bertani broth (LB; Difco) or Luria-Bertani agar (LA; Difco) medium at 37°C with aeration. When needed, erythromycin (Ery, Sigma Aldrich) was added at final concentration of 1 µg/ml. *Lactococcus lactis* LL108 strain was grown in M17 broth (Difco), supplemented with sucrose (0.5 M) and glucose (0.5% w/v) at 30°C without aeration. Tetracycline was used when required at 4 µg/ml. *Staphylococcus aureus* strains were grown at 30°C with aeration in Tryptic Soy Broth (TSB; Difco) or on Tryptic Soy Agar (TSA; Difco). Medium was supplemented when required with Ery, at 1 µg/ml. *Escherichia coli* was routinely grown in Luria-Bertani (LB; Difco) medium at 37°C or Luria-Bertani agar (LA; Difco). Ampicilin was used when required at 100 µg/ml.

**Table 1:** Bacterial strains and plasmids.

Name	Relevant Characteristics	Comments/Source/Reference
Strains		
*Streptococcus pneumoniae*		
R36A	Non-encapsulated laboratory strain.	[20]
BCSJC001	R36ApBCSJC001, Tet^r^.	[3]
BCSJC006	R36ApBCSJC006, Tet^r^.	[3]
BCSJC011	R36ApBCSJC011, Tet^r^.	This work.
BCSJC012	R36ApBCSJC012, Tet^r^.	This work.
BCSJC013	R36ApBCSJC013, Tet^r^.	This work.
BCSJC014	R36ApBCSJC014, Tet^r^.	This work.
BCSJC015	R36ApBCSJC015, Tet^r^.	This work.
BCSJC016	R36ApBCSJC016, Tet^r^.	This work.
BCSJC017	R36ApBCSJC017, Tet^r^.	This work.
BCSJC018	R36ApBCSJC018, Tet^r^.	This work.
BCSJC019	R36ApBCSJC019, Tet^r^.	This work.
BCSJC020	R36ApBCSJC020, Tet^r^.	This work.
BCSJC021	R36ApBCSJC021, Tet^r^.	This work.
BCSJC022	R36ApBCSJC022, Tet^r^.	This work.
BCSJC023	R36ApBCSJC023, Tet^r^.	This work.
BCSJC024	R36ApBCSJC024, Tet^r^.	This work.
BCSJC025	R36ApBCSJC025, Tet^r^.	This work.
BCSJC026	R36ApBCSJC026, Tet^r^.	This work.
BCSJC027	R36ApBCSJC027, Tet^r^.	This work.
BCSJC028	R36ApBCSJC028, Tet^r^.	This work.
BCSJC029	R36ApBCSJC029, Tet^r^.	This work.
BCSJC030	R36ApBCSJC030, Tet^r^.	This work.
BCSJC031	R36ApBCSJC031, Tet^r^.	This work.
BCSJC032	R36ApBCSJC032, Tet^r^.	This work.
BCSJC033	R36ApBCSJC033, Tet^r^.	This work.
BCSJC034	R36ApBCSJC034, Tet^r^.	This work.
BCSJC035	R36ApBCSJC035, Tet^r^.	This work.
BCSJC036	R36ApBCSJC036, Tet^r^.	This work.
BCSJC037	R36ApBCSJC037, Tet^r^.	This work.
BCSJC038	R36ApBCSJC038, Tet^r^.	This work.
BCSJC047	R36ApBCSJC043, Tet^r^.	This work.
BCSMH008	R36ApBCSMH007, Tet^r^.	[3]
BCSMH030	R36ApBCSLF001, Tet^r^.	[3]
BCSMH033	R36ApBCSMH002, Tet^r^.	[3]
BCSMH063	R36ApBCSMH061, Tet^r^.	This work.
*Lactococcus lactis*		
LL108	RepA^+^ MG1363, Cm^r^	[21]
BCSJC039	LL108pBCSMH002, Tet^r^.	This work.
BCSJC040	LL108pBCSJC001, Tet^r^.	This work.
*Staphylococcus aureus*		
RN4220	Restriction deficient derivative of *S. aureus* NCTC8325-4 strain capable of receiving foreign DNA	R. Novick
BCSJC041	RN4220pBCSJC039, Amp^r^; Ery^r^; LacZ^+^	This work.
BCSJC042	RN4220pBCSJC040, Amp^r^; Ery^r^; LacZ^+^	This work.
*Bacillus subtilis*		
MB24	*trpC2 metC3*	Laboratory stock
BCSJC043	MB24pBCSJC039, Amp^r^; Ery^r^; LacZ^+^	This work.
BCSJC044	MB24pBCSJC040, Amp^r^; Ery^r^; LacZ^+^	This work.
*S. pneumoniae* plasmids		
pBCSJC001	pBCSMH004 derivative, allowing expression of Citrine containing the first 10 aa of Wze at its N-terminus, Tet^r^.	[3]
pBCSJC006	pBCSJC001 derivative, TTA→CTC change in codon 4 of Wze, Tet^r^.	[3]
pBCSJC011	pBCSMH007 derivative, allowing expression of Citrine containing the first 10 aa of Wzd at its N-terminus, Tet^r^	This work.
pBCSJC012	pBCSJC037 derivative, allowing expression of Citrine containing the first 12 aa of MurM at its N-terminus, Tet^r^	This work.
pBCSJC013	pBCSJC038 derivative, allowing expression of Citrine containing the first 10 aa of MurN at its N-terminus, Tet^r^	This work.
pBCSJC014	pBCSJC001 derivative, allowing expression of Citrine containing the sequence MLEPTIAQKKL at its N-terminus, Tet^r^	This work.
pBCSJC015	pBCSJC001 derivative, allowing expression of Citrine containing the sequence MPLETIAQKKL at its N-terminus, Tet^r^	This work.
pBCSJC016	pBCSJC001 derivative, allowing expression of Citrine containing the sequence MPTILEAQKKL at its N-terminus, Tet^r^	This work.
pBCSJC017	pBCSJC001 derivative, allowing expression of Citrine containing the sequence MPTIALEQKKL at its N-terminus, Tet^r^	This work.
pBCSJC018	pBCSJC001 derivative, allowing expression of Citrine containing the sequence MPTIAQLEKKL at its N-terminus, Tet^r^	This work.
pBCSJC019	pBCSJC001 derivative, allowing expression of Citrine containing the sequence MPTIAQKLEKL at its N-terminus, Tet^r^	This work.
pBCSJC020	pBCSJC001 derivative, allowing expression of Citrine containing the sequence MPTIAQKKLEL at its N-terminus, Tet^r^	This work.
pBCSJC021	pBCSJC001 derivative, allowing expression of Citrine containing the sequence MPTIAQKKLLE at its N-terminus, Tet^r^	This work.
pBCSJC022	pBCSJC001 derivative, TTA→TTG change in codon 4 of Wze, Tet^r^.	This work.
pBCSJC023	pBCSJC001 derivative, TTA→CTT change in codon 4 of Wze, Tet^r^.	This work.
pBCSJC024	pBCSJC001 derivative, TTA→CTA change in codon 4 of Wze, Tet^r^.	This work.
pBCSJC025	pBCSJC001 derivative, TTA→CTG change in codon 4 of Wze, Tet^r^.	This work.
pBCSJC026	pBCSJC001 derivative, GAA→GAG change in codon 5 of Wze, Tet^r^.	This work.
pBCSJC027	pBCSJC001 derivative, TTA→TTG change in codon 4 of Wze and GAA→GAG change in codon 5 of Wze, Tet^r^.	This work.
pBCSJC028	pBCSJC001 derivative, TTA→CTC change in codon 4 of Wze and GAA→GAG change in codon 5 of Wze, Tet^r^.	This work.
pBCSJC029	pBCSJC001 derivative, TTA→CTA change in codon 4 of Wze and GAA→GAG change in codon 5 of Wze, Tet^r^.	This work.
pBCSJC030	pBCSJC001 derivative, TTA→CTT change in codon 4 of Wze and GAA→GAG change in codon 5 of Wze, Tet^r^.	This work.
pBCSJC031	pBCSJC001 derivative, TTA→CTG change in codon 4 of Wze and GAA→GAG change in codon 5 of Wze, Tet^r^.	This work.
pBCSJC032	pBCSJC013 derivative CTC→CTG change in codon 6 of MurN, Tet^r^.	This work.
pBCSJC033	pBCSJC013 derivative CTC→CTT change in codon 6 of MurN, Tet^r^.	This work.
pBCSJC034	pBCSJC013 derivative CTC→CTA change in codon 6 of MurN, Tet^r^.	This work.
pBCSJC035	pBCSJC013 derivative CTC→TTG change in codon 6 of MurN, Tet^r^.	This work.
pBCSJC036	pBCSJC013 derivative CTC→TTA change in codon 6 of MurN, Tet^r^.	This work.
pBCSJC037	pBCSMH002 containing *murM-Citrine*, Tet^r^.	This work.
pBCSJC038	pBCSMH002 containing *murN-Citrine*, Tet^r^.	This work.
pBCSJC043	pBCSJC001 derivative, TTA→CTC change in codon 4 of Wze, GAA→GAG change in codon 5 of Wze and ATA→AGA change in codon 6 of Wze, Tet^r^.	This work.
pBCSLF001	High-copy-number vector, contains the -10 constitutive promoter of *SigA* from *S. pneumoniae*, Tet^r^.	[4]
pBCSMH002	pBCSLF001 derivative, allows expression of Citrine fusion proteins, Tet^r^.	[4]
pBCSMH007	pBCSMH002 containing *wzd-Citrine*, Tet^r^.	[4]
pBCSMH060	pBCSMH002 containing *wchA-Citrine*, Tet^r^.	This work.
pBCSMH061	pBCSMH060 derivative allowing expression of Citrine containing the first 11 aa of WchA at its N-terminus, Tet^r^	This work.
*S. aureus/B. subtilis plasmids*		
pMAD	*E. coli* – *S. aureus* shuttle vector with a thermosensitive origin of replication for Gram-positive bacteria; Amp^r^; Ery^r^; LacZ^+^	[7]
pBCSJC039	pMAD derivative containing the -10 constitutive promoter of *SigA* from *S. pneumoniae*, the ribosome-binding site and *citrine*, Amp^r^; Ery^r^; LacZ^+^	This work.
pBCSJC040	pMAD derivative containing the -10 constitutive promoter of *SigA* from *S. pneumoniae*, the ribosome-binding site and *icitrine*, Amp^r^; Ery^r^; LacZ^+^	This work.

### DNA manipulation procedures

All plasmids used in this study are listed in Table 1 and the sequences of the primers used are listed in Table 2. PCR products and plasmid DNA were purified with kits Wizard SV Gel and PCR Clean-up System and Wizard Plus SV Minipreps, respectively (Promega). PCR fragments were amplified with Phusion High-fidelity DNA polymerase (Finnzymes). Restriction enzymes were from New England Biolabs.

**Table 2:** Primers used in this work.

Primer	Sequence 5′ → 3′	Features/Restriction sites
1	GATGAGCTCGGTACCTCGGCTG	SacI
2	GCGGAGCTCTACATCGATTTCCAAAGTGTTTTG	SacI
3	GCGGAGCTCTGCCAGAAAAATTTCCAATCC	SacI
4	GCGGAGCTCAAACTCTTCTTTCGTGAGTGTAG	SacI
5	GCGGAGCTCATATTCTAATGTGGGAATGCC	SacI
6	GCGGCTAGCCTACCTCCTTAAGCTTATTATACC	NheI
7	GCGGCTAGCATGCCGACATTGGAAATAGCAC	NheI
8	GCGGCTAGCATGCCGACACTTGAAATAGCAC	NheI
9	GCGGCTAGCATGCCGACACTAGAAATAGCAC	NheI
10	GCGGCTAGCATGCCGACACTGGAAATAGCAC	NheI
11	GCGGCTAGCATGCCGACATTGGAGATAGCAC	NheI
12	GCGGCTAGCATGCCGACACTTGAGATAGCAC	NheI
13	GCGGCTAGCATGCCGACACTAGAGATAGCAC	NheI
14	GCGGCTAGCATGCCGACACTAGAGATAGCAC	NheI
15	GCGGCTAGCATGCCGACATTAGAGATAGCAC	NheI
16	GCGGCTAGCATGCCGACACTCGAGATAGCAC	NheI
17	GCGGCTAGCATGCCGACACTCGAGAGAGCAC	NheI
18	GCGGCTAGCATGTTAGAACCGACAATAGCAC	NheI
19	GCGGCTAGCATGCCGTTAGAAACAATAGCAC	NheI
20	GCGGCTAGCATGCCGACAATATTAGAAGCACAAAAAAAACTG	NheI
21	GCGGCTAGCATGCCGACAATAGCATTAGAACAAAAAAAACTG	NheI
22	GCGGCTAGCATGCCGACAATAGCACAATTAGAAAAAAAACTG	NheI
23	GCGGCTAGCATGCCGACAATAGCACAAAAATTAGAAAAACTG	NheI
24	GCGGCTAGCATGCCGACAATAGCACAAAAAAAATTAGAACTG	NheI
25	GCGGCTAGCATGCCGACAATAGCACAAAAAAAACTGTTAGAAGAG	NheI
26	GCGGCTAGCATGGTACTAACTACATTAACG	NheI
27	GCGGCTAGCATGGTACTAACTACACTTACG	NheI
28	GCGGCTAGCATGGTACTAACTACATTGACG	NheI
29	GCGGCTAGCATGGTACTAACTACACTAACG	NheI
30	GCGGCTAGCATGGTACTAACTACACTGACG	NheI
31	GCGGGATCCGAATTCGGATCTAAAG	BamHI
32	GCGAGATCTGGATCTGGAGCTGTAATATAAAAACC	BglII
33	GAGCTGAAGGGCATCGACTT	
34	CTTGTGCCCCAGGATGTTG	
35	AATGGTTGTAGTTGCGCGCTAT	
36	AATGCTTTACCCCTATTTTCCTTTG	

### Construction of plasmids for protein expression in *S. pneumoniae*

Construction of the plasmids that allowed the expression of Citrine fused to the first 10 aminoacids of Wzd, WchA and MurN at its N-terminus was carried out by ligation of the PCR products resulting from the amplification of plasmids pBCSMH007, pBCSMH060 or pBCSJC038 with primer 1 combined with primers 2–4, respectively originating plasmids pBCSJC011, pBCSMH061 and pBCSJC013. Plasmid pBCSJC012 allowing expression of Citrine fused to the first 12 aminoacids of MurM was obtained by amplification of plasmid pBCSJC037 with primers 1/5.

For expression of Citrine fused to modified versions of the first 10 aminoacids of Wze, the pBCSJC001 plasmid was amplified with primers 6 and 20–21 and the resulting PCR products were digested and ligated producing plasmids pBCSJC014-pBCSJC021, respectively.

Construction of the plasmids encoding silent mutations in the leucine aminoacid located at position 4 of Wze, was obtained by ligation of the PCR products resulting from the amplification of plasmid pBCSJC001 with primer 6 combined with primers 7–10. The plasmids obtained in this way were named pBCSJC022–025. Plasmid pBCSJC026 encoding the GAA→GAG silent mutation in the glutamate aminoacid at position 5 of Wze was constructed by amplification of plasmid pBCSJC001 with primers 6/15.

In order to express double silent mutations in the leucine located at position 4 of Wze and glutamate at position 5 of Wze we constructed plasmids pBCSJC027- pBCSJC031. This was done by restriction and ligation of the PCR products resulting from amplification of plasmid pBCSJC001 using primers 6 and 11–14 and 6/16, respectively.

Construction of the plasmid pBCSJC043, which allowed the expression of Citrine in fusion with mutations TTA→CTC (silent mutation - leucine), GAA→GAG (silent mutation - glutamate) and ATA→AGA (mutation from isoleucine to an arginine) at positions 4–6 of Wze, was carried out by ligation of the PCR product resulting from the amplification of plasmid pBCSJC001 with primer 6 combined with primer 17.

Plasmids pBCSJC032-036 that allow Citrine expression fused, at its N-terminus, with an “i-tag” whose *murN* nucleotide sequence was modified so that it carried silent mutations in the leucine located at position 6, were obtained by restriction and ligation of the PCR product resulting from amplification of plasmid pBCSJC013 using primers 6 and 26–30.

Construction of the plasmids pBCSJC039 and pBCSJC040, which allowed the expression of Citrine and the improved form of Citrine in *S. aureus* and *B. subtilis*, was done by amplifying from pBSCMH002 or pBCSJC001 a fragment containing the −10 constitutive promoter of *SigA* from *S. pneumoniae*, the ribosome-binding site and *Citrine* or the −10 constitutive promoter of *SigA* from *S. pneumoniae*, the ribosome-binding site and *iCitrine*, respectively, with primers 31/32. The PCR fragments were purified, digested with *Bam*HI and *Bgl*II and cloned into the same sites of plasmid pMAD or pBSCMH037.

The nucleotide sequences of the modified regions of the constructed plasmids were confirmed by sequencing.

### Microscopy

*S. pneumoniae* strains were grown until early exponential phase (O. D. (600 nm) = 0.2–0.3) and observed by fluorescence microscopy on a thin layer of 1% agarose in PreC medium [16]. Images were obtained using a Zeiss Axio Observer Z1 microscope equipped with a Plan-Apochromat objective (100x/1.4 Oil Ph3; Zeiss) and a Photometrics CoolSNAP HQ2 camera (Roper Scientific). The following Semrock filter was used to visualize fluorescent signals: YFP-2427A-ZHE-ZERO for Citrine tagged proteins. After acquisition, images were analysed and cropped using Metamorph software (Meta Imaging series 7.5) and Image J software [17]. Fluorescence quantification was done using the Metamorph software by measuring the integrated fluorescence intensity in a defined region of 2 by 2 pixels and subtracting the minimum background fluorescence from every value. Quantification was performed for at least 100 cells of each strain. Statistical analysis of the fluorescence intensity data was performed using GraphPad Prism 6 (GraphPad Software, Inc.).

### RNA isolation and Quantitative Real-Time PCR

*S. pneumoniae* strains were grown in C+Y until early-exponential phase for RNA extraction. Prior to harvesting, RNAprotect Bacteria Reagent (twice the culture volume, QIAGEN) was added to the culture and the mixture was immediately vortexed for 10 sec. The cells were harvested, the pellet was frozen in liquid N_2_ and stored at −80°C overnight. The next day, the pellet was resuspended with 200 µl of sodium deoxycholate 0.25 mg/ml for 30 min at 37°C. RNA was extracted with RNeasy Mini kit (QIAGEN) and resuspended in milli-Q water. During the RNA extraction assay, an on-column DNA digestion, using 30 U of RNase-free DNase (QIAGEN), was performed for further removal of residual contaminant DNA. Total RNA was quantified using a GeneQuant*pro* Spectrophotometer (GeneQuant).

cDNA was generated from 250 ng of each RNA sample using TaqMan RT Reagents (Applied Biosystems, Branchburg, NJ, USA). The reaction mix included 5.5 mM MgCl_2_, 500 µM dNTPs, 2.5 µM random hexamers, 1× RT Buffer, 0.8 U/µl RNase Inhibitor and 1.25 U/µl MultiScribe RT in a final volume of 25 µl. The Reverse Transcription conditions were 10 min at 25°C, 15 min at 42°C and 5 min at 99°C. Quantification of Citrine expression was achieved using the LightCycler 480 (Roche, Indianapolis, In, USA), SYBR Green chemistry, and the standard curve method for relative quantification. The PCR reagents consisted of: 2× LightCycler 480 SYBR Green I Master (Roche), 400 nM of each primer, and 5 µl of sample cDNA, in a final volume of 25 µl. The thermocycling profile was: 10 min at 95°C followed by 40 cycles of 15 s at 95°C and 1 min at 60°C. qPCR primers for citrine (33 and 34) and tetracycline (35 and 36) were designed using the Primer Express (Applied Biosystems).

Optical plates included plasmid standard curves for Citrine, and duplicates of each cDNA sample. “No template” and “no RT” controls were also included in every qPCR assays. For each sample, the expression of Citrine was determined from the respective standard curve by conversion of the mean threshold cycle values, and normalization was obtained by dividing the quantity of Citrine cDNAs by the quantity of cDNA amplified within the gene encoding for the tetracycline resistance protein (used as the endogenous control), which is cloned in the same plasmid. The specificity of the amplified products was verified by analysis of the dissociation curves generated by the LightCycler 480 SYBR software based on the specific melting temperature for each amplicon. The final qPCR results were based on two independent experiments.

### Protein analysis

The analysis of the expression of the fluorescent Citrine protein linked to different N-terminal tags was done as previously described [3]. Bacterial cell aliquots of 1 ml of culture were harvested at mid-exponential growth phase. Cells were incubated at 37°C during 30 minutes in deoxicholate (0.25 mg/ml), RNase (10 mg/ml), DNase (10 mg/ml) and PMSF (1 mM). For the fluorescent protein analysis, proteins were incubated with solubilization buffer (200 mM Tris-HCl pH 8.8, 20% glycerol, 5 mM EDTA pH 8.0, 0.02% bromophenol blue, 4% SDS, 0.05 M DDT) at 37°C during 5 minutes and separated on SDS-PAGE. Gel images were acquired on a FUJI FLA 5100 laser scanner (Fuji Photo Film Co.) with 635 nm excitation and >665 nm band pass emission filter for protein molecular weight marker detection and 473 nm excitation and >510 nm band pass emission filter for Citrine detection.

For western-blot analysis, cells extracts were boiled during 5 minutes before being separated on SDS-PAGE. Proteins were transferred into a Hybond PVDF Membrane (Amersham) and probed with Living Colors Av. Peptide Antibody (Clontech) for the detection of Citrine, used at 1∶500, followed by 1∶100000 of goat anti-rabbit conjugated to horseradish peroxidase. Detection was done with ECL Plus Western Blotting Detection Reagents (Amersham).
